# Discrepancies in Drug Susceptibility Test for Tuberculosis Patients Resulted from the Mixed Infection and the Testing System

**DOI:** 10.1155/2015/651980

**Published:** 2015-05-03

**Authors:** Zaoxian Mei, Zhaogang Sun, Dapeng Bai, Yuhui Xu, Zhiling Li, Hairong Huang, Chuanyou Li, Shaofa Xu, Li Li

**Affiliations:** ^1^Department of Tuberculosis, Tianjin Haihe Hospital, Tianjin 300350, China; ^2^Tianjin Respiratory Disease Research Institute, Tianjin 300350, China; ^3^National Tuberculosis Clinical Laboratory, Beijing Chest Hospital, Capital Medical University, Beijing 101149, China; ^4^Beijing Key Laboratory in Drug Resistant Tuberculosis Research, Beijing Tuberculosis & Thoratic Tumor Research Institute, Beijing 101149, China

## Abstract

To find the potential reasons for the discrepancies in the drug susceptibility test (DST) of *M. tuberculosis* isolates, twenty paired isolates with disputed drug susceptibilities to isoniazid (INH) were selected according to the MGIT960 testing and Löwenstein-Jensen (L-J) proportion methods. Their MICs were confirmed again by broth microdilution method and by L-J proportion method. The spoligotyping results showed that, of all the 20 paired strains, 11 paired isolates belonged to the Beijing genotype and 6 paired isolates belonged to SIT1634, and that each of the remaining 3 paired isolates had two genotypes, namely, SIT1 and SIT1634. Those 3 paired isolates with different intrapair spoligotypes were further confirmed as mixed infection by the results that those three pairs of isolates with different 12 locus MIRU intrapair types and one pair carried different base pair at codon 315 (AGC versus AAC). Totally mutations in the *katG* gene were identified in 13 paired isolates. No mutations were found in the regulatory sequences and open reading frames (ORF) of the *inhA* and *ahpC* genes in any of the tested isolates. Those results showed that the different test systems and the mixed infection with particular genotypes of *M. tuberculosis* strains contributed to the drug susceptibility discrepancies.

## 1. Introduction

Performance of drug susceptibility testing (DST) to measure drug resistance is important not only before treatment, but also in the course of therapy to identify acquired resistance, especially in the areas with a high incidence of MDR-TB [1]. Conventional DST methods rely on egg-based (Löwenstein-Jensen; L-J) or agar-based (Middlebrook) media, but these are laborious and time-consuming procedures requiring 3 to 8 weeks to obtain results [2]. A number of new methods for DST, including the mycobacterial growth indicator tube (MGIT) [3], *E* test [4], and Alamar blue [5] methods, have been introduced over the last decade to detect mycobacteria rapidly and to improve their growth rates [6, 7].

The BACTEC MGIT960 method has been assessed in many countries and its degree of agreement with conventional DST methods in* M. tuberculosis* has been assessed [8–10]. Meta-analysis of published results revealed high accuracy and high predictive value associated with the use of BACTEC MGIT960 [11]. However, there are still discrepancies in the DST results obtained for different anti-TB drugs between BACTEC MGIT960 and other DST methods. The discrepancies in INH susceptibility between the MGIT960 and L-J proportion methods, for example, varied from 0% to 1% [9]; however, few investigations have been reported that addressed the possible mechanisms underlying the discrepancies between the MGIT960 system and L-J proportion methods.

Discrepancies can arise from many reasons, for example, different DST systems used, mixed infection with different* M. tuberculosis *strains, and last but not least, contamination. In this study, 20 paired isolates with disputed drug susceptibilities to INH were selected according to the MGIT960 testing and L-J proportion methods. The name of the “paired isolates” referred to the two isolates obtained separately from the cultures after the DST by MGIT960 and L-J proportion methods from the same sputum of the patient. The reasons for the DST discrepancies were analyzed by the spoligotyping and VNTR genotyping methods and drug resistance-related mutations tested in INH resistance-related genes.

## 2. Materials and Methods 

### 2.1. Strains and Antibiotics

**Figure 1:**
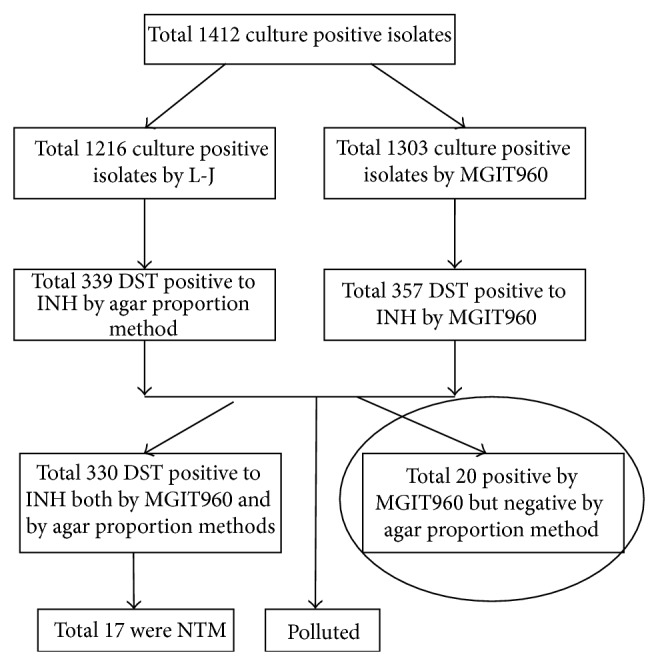
Strains selected in this experiment. A total of 1014 culture positive isolates were included in this study which were isolated in 2006. We focus on the INH as it is a very important antibiotic in curing tuberculosis. In this study of all the total 1412 culture positive isolates 1216 were positive on the L-J medium, of which 339 were resistant by L-J method to INH and 1303 isolates were positive by the MGIT960, of which 357 were resistant to INH by the MGIT960 system. Total 330 were DST positive to INH by both MGIT960 and agar proportion methods, of which 20 isolates with positive both by MGIT960 system but negative by agar proportion method were examined in this study.

A total of 20 paired* M. tuberculosis *isolates with DST discrepancies were collected in Tianjin Haihe Hospital in the year of 2006 from total 1412 isolates (Figure 1). “paired isolates” were from the the culture of the MGIT960 and L-J proportion method, respectively, which was mentioned above. Meanwhile, 96 randomly selected isolates, whose MGIT960 and agar proportion DST results were in agreement, were also collected from the same hospital. The 20 paired* M. tuberculosis *isolates were determined to be sensitive to INH using the conventional L-J proportion method (1 *μ*g/mL) [12] but resistant to INH using the BACTEC MGIT960 method (0.1 *μ*g/mL, Becton Dickinson Microbiology Systems, MD, USA) [9].* M. tuberculosis *H37Rv (ATCC 27294) obtained from the Chinese National Reference Laboratory was used as a control.

### 2.2. Determination of the MIC of INH by Middlebrook 7H9 Broth Microdilution and L-J Agar Dilution

Resazurin was used as an indicator to test the MIC of INH in the Middlebrook 7H9 broth microdilution method [13]. Briefly, a 100 *μ*L volume of Middlebrook 7H9 broth containing 0.05% Tween 80 and 10% OADC (Sigma, USA) was dispensed into the wells of a 96-well cell culture plate (Corning Coast). INH concentrations, in Middlebrook 7H9 medium, were as follows: 0.1, 0.2, 0.4, 0.8, 1.0, 1.2, 1.6, and 1.8 mg/L. Recovered isolates were collected from L-J slants and homogenized. Turbidity was adjusted to the number 1 McFarland standard (approximately 1 × 10^7^ CFU/mL) and the suspension is diluted 1 : 10 and 100 *μ*L of the dilution is added in each well that contains 100 *μ*L of the appropriate INH dilution. The final inoculum concentration was 5 × 10^4^ CFU/mL. The plates were sealed and incubated at 37°C for one week. Twenty-five microliter of 0.02% resazurin (Sigma Chem. Co., USA) solution was then added to each well and the plates were incubated for an additional 2 days. A change in color from blue to pink indicated the growth of bacteria and the MIC was read as the minimum INH concentration that prevented the color change in the presence of resazurin.

Determination of the MIC of INH using the L-J proportion method followed the protocol of the Chinese Anti-Tuberculosis Association [12]. INH concentrations used in the L-J medium were 2.0, 1.8, 1.6, 1.2, 1.0, 0.8, 0.6, 0.4, and 0.2 mg/L. About 10^5^ CFU were inoculated on the INH-containing medium slants and results were recorded after 5-6 weeks.

### 2.3. Genomic DNA Isolation, Polymerase Chain Reaction (PCR), and Sequence Analysis

Colonies were first removed from the recovering slants by scraping, resuspended in 500 *μ*L of TE (10 mM Tris, 1 mM EDTA (pH 8.0)), and killed by heating at 80°C for 30 min. The DNA extraction method, primers (from CyberSyn Co. Beijing, China), and PCR conditions were as described previously [14]. The primers were designed to amplify the* katG* gene, including the region around codon 315, the* inhA* regulatory region, the* inhA* ORF, and* oxyR-ahpC* regions (Table 1) [15, 16]. Both strands were sequenced for confirmation. Mutations were identified by BLAST comparisons with* M. tuberculosis *H37Rv as the reference (GenBank number NC_000962.3).

### 2.4. Molecular Typing by Spoligotyping and the 12-Locus MIRU Method

Spoligotyping was performed with a commercial kit (Isogen Bioscience BV, Maarssen, The Netherlands) according to the manufacturer's instructions. Amplification of the direct variant regions for spoligotyping was performed essentially as described previously [17]. Interpretation of spoligotype patterns and assignment of octal codes were based on SITVIT2 database (Pasteur Institute of Guadeloupe, Parris, France), which is an updated version of the previously released SpolDB4 database (http://www.pasteur-guadeloupe.fr:8081/SITVITDemo/tsSpoligo.jsp), as previously described [18].

The numbers of tandem repeats (TRs) at each locus in the isolates were determined on the basis of the number of whole repeats in a PCR product of the size estimated from the gel [19]. Polymerase chain reaction assays for the 12 chosen loci were repeated and compared within and between gels to ensure consistent estimation of size and TR copy number [20].

## 3. Results

### 3.1. Genotyping Analysis

Genotyping analysis can determine not only whether an infection results from transmission of the given tuberculosis isolate, but also whether the infection involves more than one strain of* M. tuberculosis*. Results from our genotyping analysis showed that 10 paired isolates belong to the Spoligotype International Type SIT1 (Beijing genotype, 000000000003771) and 6 paired isolates belong to the Spoligotype International Type SIT1634 (MANU2, 777777777723771) (Table 2), a spoligotype that was not found in the 96 randomly selected clinical isolates (Table 3). Three paired isolates were mixtures of the SIT1 and SIT1634 spoligotypes, and one pair was a mixture of SIT1 and the SIT269 (Beijing genotype, 000000000000771) spoligotypes. Compared with our set of 96 randomly selected isolates from Tianjin, only the Beijing and MANU genotypes were present and the percentage of the MANU genotype was extremely high (20 paired isolates: 15/40, 37.5%; 96 random clinical isolates: 3/96, 3.125%).

Results obtained by using the 12-locus MIRU method [19] showed that 20 pairs of isolates had 14 MIRU patterns. Both the spoligotyping and the MIRU patterns were different in the isolates named as 6, 12, and 18 pairs, individually. The isolates named as 7 pairs had different spoligotypes, but the same MIRU type (Table 2).

### 3.2. MICs of the Tested Strains

To identify the differences between the liquid Middlebrook 7H9 and L-J proportion methods in DST, we tested the MICs of each of the 16 paired INH-resistant isolates and 4 pairs of isolates which consisted of different genotypes using both Middlebrook 7H9 broth microdilution and L-J proportion methods. The MICs of all the 24 tested isolates were determined to be greater than 0.1 *μ*g/mL (0.1 to 0.6 *μ*g/mL) using the Middlebrook 7H9 broth microdilution method and greater than 0.3 *μ*g/mL (0.4 to 1.8 *μ*g/mL) using the L-J proportion method (Table 4). The MICs of 5 pairs of the tested isolates using the L-J proportion method were higher than 1 *μ*g/mL, the cutoff concentration for determining drug susceptibility in the L-J agar proportion method in this study (Table 4).

### 3.3. Sequence Analysis of the Putative INH-Target Genes

Mutations in the* katG* gene were identified in 13 paired isolates, of which each of 12 paired isolates carried the same mutations and one pair which showed a DST discrepancy by MGIT960 and L-J proportion methods carried different base pair at codon 315 (AGC versus AAC). The AGC315AAC mutation was found in 4 paired isolates, while 9 paired isolates carried the mutation AGC315ACC. The AGC315AAC and AGC 315ACC mutations were not associated with specificity to the Beijing or MANU2 genotypes among the tested isolates. Seven paired isolates did not contain mutations in the* katG* gene and no mutations were found in the regulatory sequences and open reading frames (ORF) of the* inhA* and* ahpC* genes in any of the tested isolates (Table 4). 

## 4. Discussion

Different DST methods have been developed and are used in routine clinical practice such as the conventional L-J methods and the automated MB/BacT (Organon Teknika, Turnhout, Belgium), ESPII (Difco Laboratories, Detroit, Michigan), BACTEC 9000MB (Becton Dickenson Microbiology System, Sparks, MD), and BACTEC MGIT 960 (BBL Becton Dickinson Microbiology Systems, Cockeysville, MD) systems [5, 21–23]. The DST results would be influenced by many steps of the protocol, including the culture and the DST methods. In this study, we analyzed the discrepancy of the drug susceptibility test by the MGIT and L-J methods for the isolates collected from the culture by MGIT and L-J, respectively.

Except for the median time to report the DST results the* M. tuberculosis* complex culture positivity rates were also greatly different in MGIT and L-J [24], which indicated the possible culture preference to somewhat. And the detection time, accuracy, and performance capacity are also variable by different DST methods. Studies reported that the reasons for the different performance capacity among these methods mainly resulted from the different DST systems [23, 25]. The most obvious difference is the drug concentrations used for the DST. In MGIT system, the sensitive strains were susceptible to the INH less than the 0.1 *μ*g/mL, while the concentration of the INH was 1 *μ*g/mL in L-J system in this study [12, 26, 27]. Of all the 20 paired cases 15 cases had MIC in borderlines between the MGIT and the DST methods, which was a usual reason for the discordant.

Many reports showed that there was a good concordance between DST on L-J and MGIT for INH in DST [25–27]. In this study, we still found that 20 paired isolates with the same genotypes individually showed the discrepancy in the drug susceptibilities to INH according to the MGIT960 testing and L-J proportion methods. Lawson et al. demonstrated that there was a substantial degree of agreement between the two methods, with similar INH and rifampicin DST patterns, but more frequent detection of streptomycin resistance and less frequent detection of ethambutol with L-J than MGIT-960. However, the differences were not statistically significant [25]. A multiple center evaluation showed that the discrepancies in INH susceptibility between the MGIT960 and L-J proportion methods varied from 0% to 1% [9].

Mixed infection with the different genotypes of* M. tuberculosis *in the same patient also affected the DST results even by the same testing systems [28, 29]. In this study heterogeneous genotypes were found in the isolates from each of the 4 patients. Three patients were infected by the different stains with Spoligotype International Type SIT1634 (Manu2) and Beijing genotypes and 1 patient was infected by the strains with two different Beijing genotypes. And also our test on the mutations of the putative INH-target genes,* katG*,* inhA,* and* ahpC* further confirmed one patient (number 18) with mixed infection by the heterogeneous genotypes (Table 4).

Some mycobacterial characteristics might be associated with particular genotypes. A well-known but controversial example is that the Beijing family strains of* M. tuberculosis* are often associated with relapse [30], drug resistance [31], and an increased ability to cause disease, to be transmitted within certain geographic settings [32, 33]. The isolates with particular genotypes, such as Spoligotype International Type SIT1634 (Manu2) in this study, showed higher rate of resistance in MGIT960 system than in L-J system. In this study, we found that the percentage of “MANU” genotype strains was markedly increased in paired isolates whose DST results showed discrepancies (37.5%) compared to the randomly selected clinical isolates (3.125%). An unusually high proportion of strains belonging to the “Manu” clade (27.15%) were also reported by Helal et al. [18]. Interestingly, Manu2 strains (SIT1634) have rarely been reported in Tianjin or even in China as a whole [34, 35] or in the SPOLDB4 database (excluding this study, *n* = 3, 1, from India and 2 from the USA).

In this study, all the 40 isolates were determined as resistant by MGIT and sensitive by L-J, of which twenty-seven isolates were found with mutations in* katG315 *and 13 isolates were found with no mutations in* katG315* (Table 4). Those results of the mutations found in the INH-targeted genes supported that the DST result by the MGIT was more accurate than that by the L-J, and we also found that the MICs of some isolates by L-J agar method were very higher than those in the first execution in clinic, which indicated, to some extent, that the operation needs to be improved in proportion method on L-J agar.

## 5. Conclusion

Our study confirmed that the discrepancies of the DST in* M. tuberculosis* clinical isolates did exist for INH. One of the reasons for the discrepancy is the different test systems between the BACTEC MGIT960 system and the traditional L-J proportion method. Mixed infection by the strains with MANU2 and Beijing genotype patterns could also contributed to drug discrepancies.

## Figures and Tables

**Table 1:** Primers used for PCR amplification in this study.

Gene	Forward primer, 5′-3′	Reverse primer, 5′-3′
*katG *	GCT GCT GTG GCC GGT CAA GA	CGT CCT TGG CGG TGT ATT GC
*inh*A reg	CCT CGC TGC CCA GAA AGG GA	ATC CCC CGG TTT CCT CCG GT
*inh*A ORF	GAA CTC GAC GTG CAA AAC	CAT CGA AGC ATA CGA ATA
*oxyR-ahpC *	CTG CGA CGG TGC TGG CACG	CAC GCT GCT GCG GGT GAT TGA T

MIRU and spoligotyping cluster for *M. tuberculosis* isolates		
Spoligotyping	GGT TTT GGG TCT GAC GAC	CCG AGA GGG GAC GGA AAC
MIRU02	TGG ACT TGC AGC AAT GGA CCA ACT	TAC TCG GAC GCC GGC TCA AAA T
MIRU04	GCG CGA GAG CCC GAA CTG C	GCG CAG CAG AAA CGT CAG C
MIRU10	GTT CTT GAC CAA CTG CAG TCG TCC	GCC ACC TTG GTG ATC AGC TAC CT
MIRU16	TCG GAG AGA TGC CCT TCG AGT TAG	CCC GTC GTG CAG CCC TGG TAC
MIRU20	TCG GAG AGA TGC CCT TCG AGT TAG	GGA GAC CGC GAC CAG GTA CTT GTA
MIRU23	CTG TCG ATG GCC GCA ACA AAA CG	AGC TCA ACG GGT TCG CCC TTT TGT C
MIRU24	CGA CCA AGA TGT GCA GGA ATA CAT	GGG CGA GTT GAG CTC ACA GAA
MIRU26	TAG GTC TAC CGT CGA AAT CTG TGA C	CAT AGG CGA CCA GGC GAA TAG
MIRU27	TCG AAA GCC TCT GCG TGC CAG TAA	GCG ATG TGA GCG TGC CAC TCA A
MIRU31	ACT GAT TGG CTT CAT ACG GCT TTA	GTG CCG ACG TGG TCT TGA T
MIRU39	CGC ATC GAC AAA CTG GAG CCA AAC	CGG AAA CGT CTA CGC CCC ACA CAT
MIRU40	GGG TTG CTG GAT GAC AAC GTG T	GGG TGA TCT CGG CGA AAT CAG ATA

**Table 2:** Genotypes of the 20 isolates with discrepancies in their INH DST as determined by the Middlebrook 7H9 broth microdilution and L-J agar dilution methods.

Pairs	Isolates	Spoligotyping pattern	MIRU pattern
1	2235	777777777723771	1241 2728 3422
	3010	777777777723771	1241 2728 3422

2	3195	000000000003771	1261 2718 3322
	2986	000000000003771	1261 2718 3322

3	3184	777777777723771	2261 2425 3322
	3255	777777777723771	2261 2425 3322

4	2577	000000000003771	1261 2718 3322
	549	000000000003771	1261 2718 3322

5	3478	000000000003771	1361 2618 3322
	3972	000000000003771	1361 2618 3322

**6**	322	777777777723771	1241 2728 3422
	501	000000000003771	1261 2718 3322

7	2671	000000000000771	1261 2719 3312
	1182	000000000003771	1261 2719 3312

8	2851	000000000003771	1241 2728 3422
	1563	000000000003771	1241 2728 3422

9	2566	777777777723771	1241 2728 3322
	497	777777777723771	1241 2728 3322

10	3079	777777777723771	1241 2728 3322
	2435	777777777723771	1241 2728 3322

11	3995	000000000003771	1261 2728 3322
	4835	000000000003771	1261 2728 3322

**12**	4394	000000000003771	1261 2718 3322
	4396	777777777723771	1241 2728 3422

13	4124	000000000003771	1361 2615 3322
	4198	000000000003771	1361 2615 3322

14	4192	000000000003771	2261 2615 3322
	4199	000000000003771	2261 2615 3322

15	4348	000000000003771	1261 2628 3321
	4355	000000000003771	1261 2628 3321

16	4482	777777777723771	1241 2618 3322
	1901	777777777723771	1241 2618 3322

17	4484	777777777723771	2261 2631 3321
	1914	777777777723771	2261 2631 3321

**18**	2098	777777777723771	2261 2631 3321
	2099	000000000003771	1241 2648 3322

19	2785	000000000003771	1241 2648 3422
	1554	000000000003771	1241 2648 3422

20	2789	000000000003771	1241 2648 3422
	1344	000000000003771	1241 2648 3422

Note: order of 12 MIRU loci is 2, 4, 10, 16, 20, 23, 24, 26, 27, 31, 39, and 40.

**Table 3:** Spoligotyping patterns of the 96 randomly selected *M. tuberculosis* isolates.

Number of isolates	Shared types	Spoligotyping pattern
85	Beijing (SIT1)	000000000003771
2	Beijing-like (SIT269)	000000000000771
1	Beijing-like (SIT585)	000000000000031
2	T1 (SIT261)	737777773760771
1	T1 (SIT5)	000677777760771
1	T1 (SIT353)	777777774760771
1	MANU2 (SIT53)	777777777760771
1	Manu_ancestor (SIT523)	777777777777771
1	MANU2 (SIT1195)	777767477763771
1	U (SIT1200)	703777747777771

**Table 4:** MIC of INH and the *katG*, *inhA,* and *oxyR*-*ahpC* mutations of the 20 pairs of *M. tuberculosis* isolates with DST discrepancies.

Pairs	Isolate^*^	7H9 Middlebrook (*μ*g/mL)	L-J agar (*μ*g/mL)	*katG*315	*inhA* reg	*inhA* ORF	*oxyR*-*ahpC *
1	2235	0.6	1	AAC	None	None	None
	3010	0.6	1	AAC	None	None	None
2	3195	0.1	1	AGC	None	None	None
	2986	0.1	1	AGC	None	None	None
3	3184	0.4	0.6	ACC	None	None	None
	3255	0.4	0.6	ACC	None	None	None
4	2577	0.2	0.4	AGC	None	None	None
	549	0.2	0.4	AGC	None	None	None
5	3478	0.6	1.2	ACC	None	None	None
	3972	0.6	1.2	ACC	None	None	None
6	**322**	**0.4**	**1**	ACC	None	None	None
	**501**	**0.2**	**0.6**	ACC	None	None	None
7	**2671**	**0.6**	**1.2**	AGC	None	None	None
	**1182**	**0.4**	**1**	AGC	None	None	None
8	2851	0.4	1	AAC	None	None	None
	1563	0.4	1	AAC	None	None	None
9	2566	0.4	1	ACC	None	None	None
	497	0.4	1	ACC	None	None	None
10	3079	0.6	1.4	AGC	None	None	None
	2435	0.6	1.4	AGC	None	None	None
11	3995	0.4	1	ACC	None	None	None
	4835	0.4	1	ACC	None	None	None
12	**4394**	**0.4**	**0.8**	ACC	None	None	None
	**4396**	**0.4**	**0.8**	ACC	None	None	None
13	4124	1	1.4	AAC	None	None	None
	4198	1	1.4	AAC	None	None	None
14	4192	0.4	1	ACC	None	None	None
	4199	0.4	1	ACC	None	None	None
15	4348	0.2	0.8	ACC	None	None	None
	4355	0.2	0.8	ACC	None	None	None
16	4482	0.4	1	AGC	None	None	None
	1901	0.4	1	AGC	None	None	None
17	4484	1	1.8	ACC	None	None	None
	1914	1	1.8	ACC	None	None	None
18	**2098**	**0.4**	**1**	**AGC**	None	None	None
	**2099**	**0.4**	**1**	**AAC**	None	None	None
19	2785	0.4	1	AAC	None	None	None
	1554	0.4	1	AAC	None	None	None
20	2789	0.2	0.6	AGC	None	None	None
	1344	0.2	0.6	AGC	None	None	None

Note: *katG*315 is the predominant mutation. The wild type is AGC.

^*^16 isolates with consistent genotype in pair and 4 pairs of isolates (**bold**) with different genotypes in pair.
